# ERN GENTURIS tumour surveillance guidelines for individuals with neurofibromatosis type 1

**DOI:** 10.1016/j.eclinm.2022.101818

**Published:** 2023-01-13

**Authors:** Charlotte Carton, D. Gareth Evans, Ignacio Blanco, Reinhard E. Friedrich, Rosalie E. Ferner, Said Farschtschi, Hector Salvador, Amedeo A. Azizi, Victor Mautner, Claas Röhl, Sirkku Peltonen, Stavros Stivaros, Eric Legius, Rianne Oostenbrink, ERN GENTURIS NF1 Tumour Management Guideline Group, Joan Brunet, Frank Van Calenbergh, Catherine Cassiman, Thomas Czech, María José Gavarrete de León, Henk Giele, Susie Henley, Conxi Lazaro, Vera Lipkovskaya, Eamonn R. Maher, Vanessa Martin, Irene Mathijssen, Enrico Opocher, Ana Elisabete Pires, Thomas Pletschko, Eirene Poupaki, Vita Ridola, Andre Rietman, Thorsten Rosenbaum, Alastair Santhouse, Astrid Sehested, Ian Simmons, Walter Taal, Anja Wagner

**Affiliations:** a Laboratory for Neurofibromatosis Research, Department of Human Genetics, University of Leuven, KU Leuven, Belgium; b Manchester Centre for Genomic Medicine, Division of Evolution and Genomic Sciences, University of Manchester, MAHSC, St Mary's Hospital, Manchester University Hospitals NHS Foundation Trust, Manchester, UK; c Clinical Genetics Department, Hospital Germans Trias I Pujol, Barcelona, Spain; d Universitätsklinikum Hamburg-Eppendorf, Hamburg, Germany; e Neurofibromatosis Centre, Department of Neurology, Guy's & St Thomas' NHS Foundation Trust, London, UK; f Sant Joan de Déu, Barcelona Children's Hospital, Barcelona, Spain; g Division of Neonatology, Pediatric Intensive Care and Neuropediatrics, Department of Pediatrics and Adolescent Medicine, Medical University of Vienna, Austria; h NF Kinder, Austria; i University of Turku and Turku University Hospital, Turku, Finland; j Sahlgrenska University Hospital and Sahlgrenska Academy, University of Gothenburg, Sweden; k Academic Unit of Paediatric Radiology, Royal Manchester Children's Hospital, Central Manchester University Hospitals NHS Foundation Trust, Manchester, UK; l Geoffrey Jefferson Brain Research Centre, Northern Care Alliance NHS Group, University of Manchester, Manchester Academic Health Science Centre, Manchester, UK; m University Hospital Leuven, Department of Human Genetics, University of Leuven, KU Leuven, Belgium; n ENCORE-NF1 Expertise Center, ErasmusMC-Sophia, Rotterdam, the Netherlands

**Keywords:** Guideline, Neurofibromatosis type 1, Tumour predisposing syndrome, Management

## Abstract

**Background:**

Neurofibromatosis type 1 (NF1) is a multisystem genetic disorder, predisposing development of benign and malignant tumours. Given the oncogenic potential, long-term surveillance is important in patients with NF1. Proposals for NF1 care and its specific manifestations have been developed, but lack integration within routine care. This guideline aims to assimilate available information on NF1 associated tumours (based on evidence and/or expert opinion) to assist healthcare professionals in undertaking tumour surveillance of NF1 individuals.

**Methods:**

By comprehensive literature review, performed March 18th 2020, guidelines were developed by a NF1 expert group and patient representatives, conversant with clinical care of the wide NF1 disease spectrum. We used a modified Delphi procedure to overcome issues of variability in recommendations for specific (national) health care settings, and to deal with recommendations based on indirect (scarce) evidence.

**Findings:**

We defined proposals for personalised and targeted tumour management in NF1, ensuring appropriate care for those in need, whilst reducing unnecessary intervention. We also incorporated the tumour-related psychosocial and quality of life impact of NF1.

**Interpretation:**

The guideline reflects the current care for NF1 in Europe. They are not meant to be prescriptive and may be adjusted to local available resources at the treating centre, both within and outside EU countries.

**Funding:**

This guideline has been supported by the European Reference Network on Genetic Tumour Risk Syndromes (ERN GENTURIS). ERN GENTURIS is funded by the 10.13039/501100000780European Union. DGE is supported by the Manchester 10.13039/100015250NIHR10.13039/100014461Biomedical Research Centre (IS-BRC-1215-20007).

**Research in context**

**Evidence before this study**

Given the potential for tumour development, long-term surveillance is of utmost importance both in children and in adults with NF1. New proposals for care of individuals with NF1 have been developed as there are several disease specific issues that differ from the general approach in sporadic tumours, but need to be integrated within routine care. Patients with NF1 do not have access to the same level of care in all countries, and treatment varies across different institutions.

**Added value of this study**

We defined recommendations for tumour management in NF1 in a personalised and targeted approach. Appropriate care for those NF1 patients in need was balances versus reducing unnecessary investigations for those without NF1 complications. We also incorporated tumour-related psychosocial and quality of life aspects.

**Implications of all the available evidence**

Based on all available evidence, we defined recommendations for tumour management in NF1, balancing appropriate care for those in need versus unnecessary treatments for those without complications, incorporating tumour related psychosocial and quality of life aspects. Given the low prevalence of the condition, its many potential manifestations and rare complications, decisions about management should always include discussion with the local multidisciplinary teams including an NF1 experts. The guidelines are not meant to be prescriptive and may be adjusted according to the local health care system.

## Introduction

Neurofibromatosis type 1 (NF1) is an autosomal dominant inherited disorder with an estimated birth incidence of one in 2000–2500.^1^ Approximately half of the patients with NF1 have inherited the disorder from their parents, while the other half acquired a *de novo* pathogenic variant in the *NF1* gene. NF1 can be diagnosed using the revised diagnostic criteria which include the presence of two of the following: ≥ six café-au-lait-macules, skinfold freckling, neurofibromas, optic pathway glioma (OPG), Lisch nodules or choroid abnormalities, bone dysplasia, the presence of a heterozygous pathogenic *NF1* variant, and the presence of a parent with NF1.^2^

NF1 predisposes to the development of benign and malignant tumours such as peripheral nerve sheath tumours, brain tumours, phaeochromocytomas, paragangliomas, gastrointestinal stromal tumours (GISTs), breast cancer, glomus tumours of the digits and a specific type of leukaemia.^3^^,^^4^ An additional common manifestation associated with NF1 is neurocognitive impairment, such as learning disability, attention-deficit hyperactivity disorder, autism spectrum disorder, behavioural problems and specific learning difficulties.^5^, ^6^, ^7^, ^8^, ^9^ Furthermore, patients with NF1 may experience other organ specific manifestations such as vascular changes (amongst others moyamoya type vasculopathy^10^^,^^11^), and several skeletal defects.^12^, ^13^, ^14^ Therefore, it is a disease that cuts across multiple disciplines and considerably affects quality of life.^1^^,^^15^

NF1 affects the individual from birth, thereby predisposing the patient to multisystem tumour complications from a very young age on. The lifetime risk of cancer in patients with NF1 was estimated to be 59.6%, compared to 30.8% in the general population.^16^ The median age of cancer diagnosis in patients with NF1 is 39 years. However, the standardised incidence ratio for cancer in children and women <30 years with NF1 is especially high.^16^ The life expectancy of patients with NF1 is generally decreased by 8–15 years, mainly due to malignancies.^17^, ^18^, ^19^

Given the potential for tumour development, long-term surveillance is of utmost importance both in children and in adults, and should be performed by clinicians who understand the condition and can provide lifelong care. New proposals for care of individuals with NF1 have been developed as there are several disease specific issues that differ from the general approach in sporadic tumours, but need to be integrated within routine care. It is important not to delay assessment for patients who are at risk of serious complications; monitoring, surveillance and management of individuals with NF1 requires a multidisciplinary approach and specific guidance adapted to the specific risks and natural history of the disease.

Patients with NF1 do not have access to the same level of care in all countries, and treatment varies across different institutions. This guideline aims to improve quality of care by presenting information to assist healthcare professionals in tumour surveillance and management of individuals with NF1 in an attempt to provide equity in healthcare for all patients with NF1.

The guideline specifically aims to integrate available evidence-based and or expert opinion-based information to assist healthcare professionals in regard to tumour surveillance of individuals with a confirmed NF1 diagnosis. This guideline has been written by members of the European Reference Network (ERN) for Genetic Tumour Risk Syndromes (GENTURIS) directed at member states of the European Union, but probably reflects an approach that could be applied on a broader scale. Recommendations are not prescriptive and may be adapted to the local health care system.

This guideline addresses surveillance for tumour types associated with NF1,^16^^,^^20^ offers guidance on the imaging modalities for surveillance, on the age to start and the interval between assessments. Moreover, recognising that there are potential associated neurocognitive deficits and psychosocial needs we included specific approaches/guidelines required for this complex condition.

Given the low prevalence of the condition, its’ many potential manifestations and complications, decisions about management should be discussed in multidisciplinary teams including an NF1 expert.

## Methods

The ERN GENTURIS Guideline Group for Neurofibromatosis Type 1 (NF1 Tumour Management Guideline Group) was established by experts in NF1 from 11 countries (n = 32), encompassing the clinical care of the disease spectrum in children and adults as well as patient representatives (n = 6). The NF1 Tumour Management Guideline Group was supported by a Core Working Group (n = 14) which comprised ERN GENTURIS healthcare provider members from different Member States and other experts who are recognised experts and specialised in clinical practice and/or in the diagnosis and tumour management of NF1.

In order to recruit members for the NF1 Tumour Management Guideline Group and Core Working Group, a request for willing participants was made within ERN GENTURIS. ERN GENTURIS members with expertise in NF1 and additional non-ERN GENTURIS European NF1 experts were selected for the Core Working Group (requirement to have at least 2 ERN GENTURIS health care providers from at least 2 Member States with expertise in the ERN GENTURIS thematic group 1 Neurofibromatosis). Afterwards, the Core Working Group suggested European experts in the field (colleagues) for the NF1 Tumour Management Guideline Group. The external experts/Delphi participants were all suggested by members of the NF1 Tumour Management Guideline Group. When representation from specific European countries was low, ERN GENTURIS national coordinators of the respective country were contacted and encouraged to suggest local experts. During the selection of the final group of experts we took into account the coverage of all specialists and all European countries. However, expertise coverage was leading the selection. Patient representatives were also first recruited within ERN GENTURIS, and they in turn suggested other representatives.

For all manifestations, we discussed (i) what clinical screening is appropriate for detecting tumours, (ii) what imaging screening is useful for detecting tumours and how this differs in NF1 from the general population, (iii) what method and monitoring interval is used if a tumour is diagnosed (if applicable); (iv) what the indication is for treatment and whether the type of treatment in NF1 is different from the general population. In addition, we discussed and advised on the role of imaging techniques in NF1 management such as optic coherence tomography (OCT) and whole-body magnetic resonance imaging (WB-MRI) that are becoming more routinely available. Finally, the psychosocial support that people with NF1 need during the surveillance, monitoring and/or treatment of a specific NF1 associated tumour is addressed. The discussions were held during monthly meetings via videoconferencing with members of the Core Working Group. Pending on the load and level of evidence for each manifestation, recommendations for one or more manifestations were addressed per meeting. Also (revisions of) recommendations for one manifestation could be addressed in multiple meetings.

**Fig. 1:**
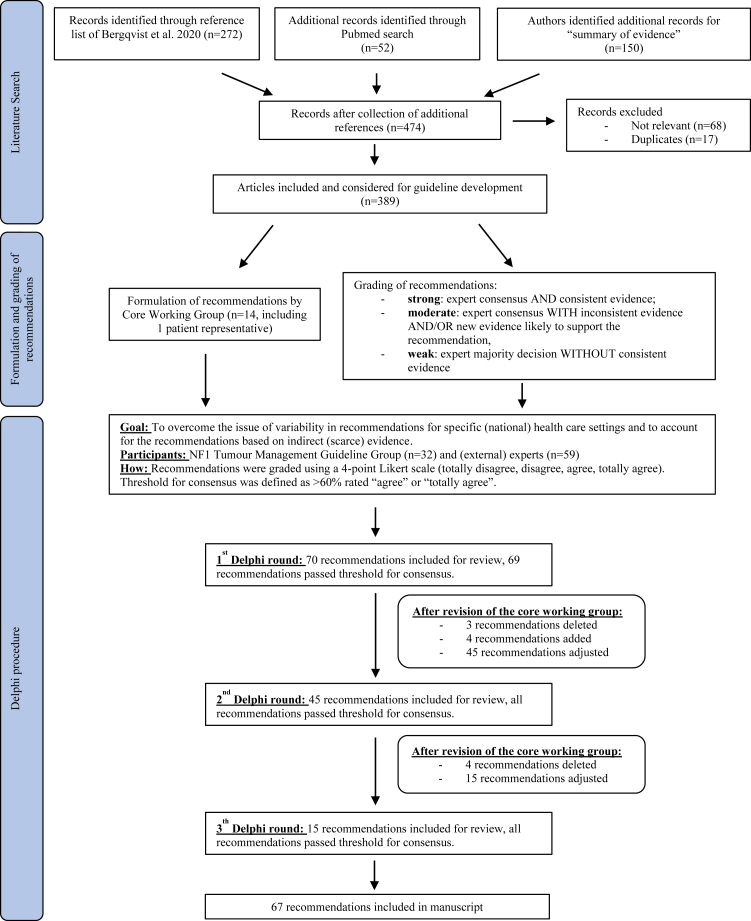
Flowchart of the search results, formulation and grading of the recommendations and Delphi procedure.

The guideline is a comprehensive literature review based on an existing literature review of Bergqvist et al*.*^21^ As this review contained literature up to 2013, additional searches were performed for each section of this guideline using the following terms in PubMed: (Neurofibromatosis Type 1 [title/abstract] OR NF1 [title/abstract]) AND optic pathway glioma [title/abstract] OR non-optic glioma [title/abstract] OR malignant peripheral nerve sheath tumour [title/abstract] OR orbital plexiform neurofibroma [title/abstract] OR periorbital plexiform neurofibroma [title/abstract] OR plexiform neurofibroma [title/abstract] OR cutaneous neurofibroma [title/abstract] OR gastrointestinal stromal tumours [title/abstract] OR phaeochromocytoma [title/abstract] OR breast cancer [title/abstract] OR glomus tumours of the digits [title/abstract] OR Juvenile myelomonocytic [title/abstract]. The literature search in PubMed was performed on the 18th of March, 2020. Secondary to this systemic literature search, authors identified additional records for “summary of evidence” (n = 150) based on their own expertise or snowballing of reference lists of publications. Important new references were added up until completion of the guideline document. After collecting additional references a total of 474 records were identified (Fig. 1). Seventeen duplicates were removed and 68 papers were excluded due to not being relevant to surveillance, follow-up and management of tumours in people with NF1. A total of 389 published articles were included in the development of the guideline (either providing background information and epidemiology or specifically addressing recommendations). For this current manuscript, only references underlying the recommendations (n = 119) were included in the reference list. As is typical for many rare diseases, the volume of peer reviewed evidence available to consider for these guidelines was small and came from a limited number of scientific publications, which frequently reported small series of patients.

To balance the weight of both published evidence and quantify the wealth of expert experience and knowledge, ERN GENTURIS uses the following scale to grade the recommendation: (i) strong: expert consensus AND consistent evidence; (ii) moderate: expert consensus WITH inconsistent evidence AND/OR new evidence likely to support the recommendation, and (iii) weak: expert majority decision WITHOUT consistent evidence (Fig. 1).

To overcome the issue of variability in recommendations for specific (national) health care settings and to account for the recommendations based on indirect (scarce) evidence, we applied a modified Delphi procedure. Experts in this exercise included the members of the NF1 Tumour Management Guideline Group, as well as an additional 59 (external) experts identified by the Guideline Group. In each Delphi round the threshold for consensus was defined by a majority of the survey participants agreeing with the recommendation (>60% rated “agree” or “totally agree”). Recommendations were graded using a 4-point Likert scale (totally disagree, disagree, agree, totally agree) and a justification for the given rating was optional in a free text format. The grading and justification were given independently in each Delphi round in order to avoid participant bias or response bias between participants. Even if consensus was met recommendations were still modified if a higher consensus was thought achievable after review of the written responses.

An initial set of 70 recommendations developed by the ERN GENTURIS NF1 Tumour Management Core Working Group were selected to be part of the Delphi procedure (Fig. 1). The facilitator of the Delphi survey provided an anonymised summary of the experts’ decisions as well as the reasons they provided for their responses. After each Delphi round, the anonymised results (consensus statistics) were distributed among the participants. The Core Working Group discussed the anonymised summary of comments given to all recommendations in each round and decided to delete, add, or adjust the recommendations accordingly. These new sets of recommendations were then iteratively subjected to subsequent rounds of the Delphi survey. After three Delphi rounds, 67 recommendations were included in the final guideline. All recommendations passed the threshold for consensus and reached similar or higher percentage of agreement.

## Website

The complete guidelines can be downloaded from the ERN GENTURIS website: https://www.genturis.eu.

## Role of funding

This guideline has been supported by the European Reference Network on Genetic Tumour Risk Syndromes (ERN GENTURIS). ERN GENTURIS is funded by the 10.13039/501100000780European Union. DGE is supported by the Manchester 10.13039/100015250NIHR 10.13039/100014461Biomedical Research Centre (IS-BRC-1215-20007).

## Results

Recommendations in this guideline address surveillance, follow-up and management of NF1-asociated tumours. During the Delphi-survey we noticed that the definition of surveillance is not always clear as it can mean identifying tumours in high-risk patients as well as follow-up of patients in whom a tumour was detected. Therefore, we decided to use the term “clinical assessment” or “imaging screening” throughout the document. Clinical assessment is part of long-term surveillance and includes history taking and physical examination by a qualified and experienced clinician. Imaging screening implies imaging-based assessment for potential tumours that are not yet known to be present. Monitoring is used for the follow-up of tumours that are known to be present, but which do not need immediate treatment, and which may have a variable course.

### Guideline summary and general approach

All the recommendations are summarised in Table 1.

**Table 1:** Summary of the surveillance protocol for tumour screening/identification in individuals with Neurofibromatosis type 1.

Tumour	Surveillance	Interval	From age (years)/indication	Strength^a^	Reference to table in article
Optic pathway glioma	Clinical assessment:1. Visual assessment2. Fundoscopy3. Visual fields4. Optic coherence tomography	1-3: At least yearly 4: When feasible	0–8	1. Strong2. Strong3. Moderate4. Moderate	Table 3
Optic pathway glioma	Visual screening	Yearly	8 – transition adolescence to adult	Moderate	Table 3
Brain or spine glioma	Patient history/Examination signs of brain tumours	Every visit	All ages	Moderate	Table 4 for children and Table 5 for adults
Cutaneous neurofibroma	Clinical examination	Every visit	All ages	Strong	Table 6
Plexiform neurofibroma	Clinical examination	Every visit	All ages	Moderate	Table 7
Plexiform neurofibroma	Whole-body MRI	Once	Transition adolescence- adult	Weak	Table 7
Orbital & Periorbital Plexiform neurofibroma	Clinical assessment, refraction error, vision fields, ocular motility	Every visit	All ages	Strong	Table 8
Malignant peripheral nerve sheath tumour + Atypical neurofibromateous neoplasm of uncertain biologic potential	Clinical examination + history taking	Every visit	All ages	Strong	Table 9
Malignant peripheral nerve sheath tumour + Atypical neurofibromateous neoplasm of uncertain biologic potential	Regional MRI combined with ^18^FDG PET MRI or ^18^FDG PET CT	On indication	Suspicion for malignancy	Moderate	Table 9
Juvenile myelomonocytic leukaemia	As part of normal clinical routine: patient history and physical examination	Every visit	<12	Moderate	Table 10
Breast cancer	MRI or mammography being second best alternative when MRI is not available	Yearly	30–50	Moderate	Table 11
Breast cancer	Breast screening per national guideline for the general population	Breast screening per national guideline for the general population	>50	Moderate	Table 11
Phaeochromocytoma and paraganglioma	Biochemical screening	On indication	Raised blood pressure	Moderate	Table 12
Phaeochromocytoma and paraganglioma	Biochemical screening	On indication	Pregnant women and consider if elective surgery requiring general anaesthesia	Weak	Table 12
Glomus tumours of the digits	Screening for symptoms and visual inspection	Every visit	All ages, clinical suspicion	Moderate (Age, weak)	Table 13
Gastrointestinal stromal tumour	Clinical examination + history taking	Every visit	Adolescence and adults	Moderate	Table 14
Gastrointestinal stromal tumour	Abdominal MRI or CT	On indication	Clinical suspicion of presence based on symptoms	Moderate	Table 14
Psychosocial needs	Psychosocial wellbeing and neuropsychological functioning	Every visit	All ages	Weak	Table 15

Note. MRI = magnetic resonance imaging; ^18^FDG PET MRI = 18F-fluorodeoxyglucose positron emission tomography magnetic resonance imaging; ^18^FDG PET CT = 18F-fluorodeoxyglucose positron emission tomography computed tomography; CT = computed tomography.

a To balance the weight of both published evidence and quantify the wealth of expert experience and knowledge, ERN GENTURIS uses the following scale to grade the recommendation:

**Table:** 

Strength	Grading of recommendation
Strong	Expert consensus AND consistent evidence
Moderate	Expert consensus WITH inconsistent evidence AND/OR new evidence likely to support the recommendation
Weak	Expert majority decision WITHOUT consistent evidence

Expert consensus (an opinion or position reached by a group as whole) or expert majority decision (an opinion or position reached by the majority of the group) is established after reviewing the results of the modified Delphi approach within the Core Working Group.

NF1 associated tumours can be age dependent. Some NF1 associated tumours have an increased risk and prevalence in childhood such as OPGs, and others are seen more commonly in adults such as malignant peripheral nerve sheath tumours (MPNSTs), phaeochromocytomas and GISTs. However, in rare occasions, MPNSTs do occur in children, with a poor prognosis compared to children without NF1.^22^^,^^23^ Rhabdomyosarcoma and neuroblastoma are also paediatric neoplasms associated with NF1, but due to their extremely low prevalence, screening for these neoplasms is currently not recommended.^3^ Based on these risks for NF1 tumour complications, this guideline recommends systematic clinical assessment by NF1 experts at regular intervals as soon as NF1 is diagnosed or suspected (Table 1, Table 2, Table 3).

**Table 2:** Guideline Recommendations for general approach.

General approach		
No	Recommendations	Strength
1	Based on the risk of occurrence of tumour complications in NF1, systematic clinical assessment by NF1 experts at regular intervals is advised: - with a minimum of annually in children up to 10 years - with a minimum of once every two years in children older than 10 years - with a minimum of once every 3 years in adults During transition from adolescence to adulthood more frequent systematic clinical assessment (than the above mentioned) may be warranted.	weak

Note. NF1 = Neurofibromatosis type 1.

**Table 3:** Guideline recommendations for optic pathway glioma.

Optic pathway glioma		
No	Recommendations	Strength
1	Clinical assessment for OPG should begin immediately after diagnosis or suspicion of NF1 in childhood. Baseline ophthalmology assessment should be done at presentation whatever the age.	strong
2	Clinical assessment for OPG should take the form of examination by trained paediatric ophthalmologists or neuro-ophthalmologists or equivalent with experience in the assessment of NF1 related visual changes.	strong
3	Clinical assessment for OPG should include age-appropriate assessment of visual acuity, visual fields, pupillary testing, eye movements, and optic disc appearance.	strong
4	Assessment of retinal nerve fibre layer and retinal ganglion cell layer by optic coherence tomography is helpful and should be conducted whenever feasible.	moderate
5	For children until the age of 8 years without known OPG, ophthalmological assessment (see recommendation 1–3) should be repeated at least every year (every six months if feasible).	moderate
6	In children >8 years without known OPG formal annual visual screening is advised until adulthood. Diagnostic evaluation by an ophthalmologist is also indicated in those with new visual symptoms.	moderate
7	Imaging for OPG with MRI should be performed in people where ophthalmological examination is suggestive for OPG and in children older than 2 years with repeated inconclusive or unreliable ophthalmological exam, e.g. due to age or attention deficit. Abnormal, inconclusive or unreliable ophthalmological exam should be repeated within a short timeframe.	strong
8	Any patient with NF1 diagnosed with an asymptomatic OPG should receive a referral to a unit with expertise (e.g. paediatric, ophthalmology, and/or neuro-oncology) in the monitoring and management of NF1-OPG.	moderate
9	Any patient with NF1 diagnosed with a symptomatic OPG should receive an urgent referral to a unit with expertise (e.g. paediatric, ophthalmology, and/or neuro-oncology) in the management of NF1-OPG.	strong

Note. OPG = optic pathway glioma; NF1 = Neurofibromatosis type 1; MRI = magnetic resonance imaging.

For children up to ten years annual assessments to detect complications of NF1 are advised. Children older than ten years require clinical assessments *at least* once every two years (Table 1, Table 2). These annual or two-yearly assessments of paediatric patients with NF1 should include: patient history taking and clinical examination for signs/symptoms associated with paediatric NF1 tumour manifestations (Table 1). In addition, children until the age of eight years without a known OPG, should be examined by trained paediatric ophthalmologists, neuro-ophthalmologists, or someone with equivalent experience in the assessment of NF1 related visual changes. In children older than eight years without known OPG formal annual visual screening is advised until adulthood. When a plexiform neurofibroma is detected, the lesion should be monitored (Table 1, Table 7). Parents can be questioned regarding occurrence or any change in existing pain, growth or texture of the lesion. In case of pain, change in texture or sudden increase in growth, the plexiform neurofibroma should be evaluated for possible malignant transformation. During the transition from adolescence to adulthood more frequent systematic clinical assessment may be warranted. Monitoring for plexiform neurofibromas should exist of WB-MRI imaging at least once at transition from childhood to adulthood to evaluate internal tumour burden as a predictor for MPNSTs. In the absence of internal plexiform neurofibromas on WB-MRI, further monitoring using WB-MRI is not recommended. Localised guided imaging assessment is required at all ages in case of symptoms evocative of MPNST formation at clinical assessment (Table 7).

**Table 4:** Guideline Recommendations for non-optic pathway glioma (non-OPG: low- or high-grade brain or spine glioma) in children.

Non-optic pathway glioma in children		
No	Recommendations	Strength
1	Families with children with NF1 should be educated about possible symptoms and signs of brain tumours.	moderate
2	Clinical assessment should take the form of patient history taking and examination for signs of brain tumours (amongst others new onset or change in seizures, unusual or concerning headache, endocrine problems related to hypothalamic dysfunction, focal neurological deficits, neuropsychological deficits) and should be repeated at every clinical visit from diagnosis.	moderate
3	Routine diagnostic imaging screening for non-OPG, in children who are well (see previous recommendation), is not indicated. However, in a child with clinical concern for a brain tumour, e.g. in the presence of symptoms or endocrine dysfunction, then investigative imaging should be recommended.	moderate
4	Symptomatic non-OPG in children with NF1 should be treated by the same care pathway as sporadic non-OPG in children without NF1. A multidisciplinary team should guide on appropriate therapeutic agents in the setting of NF1. Radiotherapy should be avoided, if at all possible, and is not indicated in low-grade glioma, whilst recognising that it may be required as an important treatment option in the setting of high-grade glioma.	moderate

Note. NF1 = Neurofibromatosis type 1; OPG = optic pathway glioma.

**Table 5:** Guideline Recommendations for non-optic pathway glioma (non-OPG: low- or high-grade brain or spine glioma) in adults.

Non-optic pathway glioma in adults		
No	Recommendations	Strength
1	Patients with NF1, their carers and primary care physicians should be educated about possible symptoms and signs of brain tumours in a manner appropriate to the individual patient.	moderate
2	Clinical assessment should take the form of examination for signs of brain tumours (amongst others new onset or change in seizures, new onset, unusual or concerning headache, endocrine problems related to hypothalamic dysfunction, focal neurological deficits, neuropsychological deficits) at every clinical visit.	moderate
3	Imaging screening for gliomas should be considered at the age of transition from childhood to adulthood for all patients with NF1 and should take the form of brain MRI with contrast. Imaging investigation should also be undertaken after new associated symptoms (amongst others new onset or change in seizures, new onset, unusual or concerning headache, endocrine problems related to hypothalamic dysfunction, focal neurological deficits, neuropsychological deficits) or positive physical examination findings.	moderate
4	Incidental detected gliomas should be followed up with imaging like sporadic incidental detected gliomas, with a first interval of 3 months, and if stable asymptomatic disease, intervals can be prolonged.	weak
5	Non-OPG in adults with NF1 should be managed and treated through the same care pathways as sporadic non-OPG. A multidisciplinary team should guide on appropriate therapeutic agents in the setting of NF1. Radiotherapy should be avoided if at all possible, and is not indicated in low-grade glioma, whilst recognising that it may be required as an important treatment option in the setting of high-grade glioma.	strong

Note. NF1 = Neurofibromatosis type 1; MRI = magnetic resonance imaging; OPG = optic pathway glioma.

**Table 6:** Guideline Recommendations for cutaneous neurofibroma.

Cutaneous neurofibroma		
No	Recommendations	Strength
1	Clinical assessment consisting of visual inspection and palpation should begin when NF1 is diagnosed and should be repeated at every clinical visit.	strong
2	Discomfort for the patient should be the primary indication for treatment. With regard to aesthetic considerations the impacts are unique to each individual and each health system has its own criteria and thresholds for intervention, so this should be considered on a case-by-case with discussion between the treating team and person with NF1.	weak
3	Removal should be by laser, surgery, electrodessication or radiofrequency ablation. If multiple tumours are removed, histological assessment of all clinically obvious small cutaneous neurofibroma is not necessary.	moderate
4	Given the burden of the visible manifestations in NF1 with cutaneous neurofibroma, patients with cutaneous neurofibroma should be offered psychological support (please see recommendations in the psychosocial needs, Table 15).	weak

Note. NF1 = Neurofibromatosis type 1.

**Table 7:** Guideline Recommendations for plexiform neurofibroma.

Plexiform neurofibroma		
No	Recommendations	Strength
1	Clinical assessment should be by observation, palpation and neurological examination and should be performed by clinicians with NF1 expertise. Photography or video of the plexiform neurofibroma can be useful adjuncts.	moderate
2	Clinical assessment for plexiform neurofibroma should start at diagnosis or birth and should be carried out at every clinical visit.	moderate
3	Imaging by whole body MRI (WB-MRI) to monitor for plexiform neurofibromas should be performed at least at transition from childhood to adulthood to evaluate internal tumour burden as a predictor for the development of malignant peripheral nerve sheath tumour (MPNST) risk. WB-MRI assessment at higher frequency may be considered for patients at high risk for MPNST.	weak
4	The frequency of repeat imaging should be determined on an individual basis guided by the multidisciplinary team assessment of the level of risk for the individual. Increased assessment may be considered for patients with high risk for MPNST. In absence of internal neurofibromas at WB-MRI at transition age to adulthood clinical assessment only is required.	moderate
5	Clinical monitoring of plexiform neurofibromas should start when first detected and repeated during each visit.	moderate
6	Symptomatic plexiform neurofibromas require increased monitoring at shorter intervals for ANNUBP/MPNST. With careful judgement, it is appropriate to use ^18^FDG PET MRI (preferred) or ^18^FDG PET CT (if ^18^FDG PET MRI is not available) combined with clinical assessment and MRI in the diagnostic process, prior to discussing the need for biopsy.	moderate
7	For symptomatic plexiform neurofibroma^a^, surgery is the only treatment that can potentially cure the tumour. Plexiform neurofibroma surgery should be considered.	moderate
8	If part of standard national care, MEK-inhibitors may be considered as treatment option for symptomatic plexiform neurofibroma^a^, and inoperable symptomatic plexiform neurofibromas.	moderate
9	Management of plexiform neurofibroma should be decided upon and performed by a multidisciplinary team with expertise in NF1.	weak
10	Given the burden of having a potential risk of malignancy and visible manifestation in patients with NF1 with plexiform neurofibroma, people with plexiform neurofibromas should be offered psychological support in decisions of management (please see recommendations in the psychosocial needs, Table 15).	weak

NF1 = Neurofibromatosis type 1; WB-MRI = whole-body magnetic resonance imaging; MPNST = malignant peripheral nerve sheath tumour; ANNUBP = Atypical neurofibromateous neoplasm of uncertain biologic potential; ^18^FDG PET MRI = 18F-fluorodeoxyglucose positron emission tomography magnetic resonance imaging; ^18^FDG PET CT = 18F-fluorodeoxyglucose positron emission tomography computed tomography; MRI = magnetic resonance imaging; MEK = mitogen-activated protein kinase.

a symptomatic plexiform neurofibromas are: persistent pain not responsive to treatment in regional pain centre, disfigurement, functional deficit or potential deficit including neurological deficit, bladder, bowel, respiratory or swallowing problems or haemorrhage.

In adulthood the risk of developing MPNSTs, atypical neurofibromatous neoplasms of uncertain biologic potential (ANNUBPs, formally called atypical neurofibromas), cutaneous neurofibromas, breast cancer, GISTs, glomus tumours of the digits, phaeochromocytomas and paragangliomas increases.^16^ This guideline recommends that adults with NF1 should be assessed clinically, at least once every three years (Table 1, Table 2). Every visit should consist of history taking, examination for signs/symptoms of brain tumours or MPNSTs, examination of the skin for cutaneous neurofibromas, and screening for/monitoring of plexiform neurofibromas. Repeat WB-MRI imaging for monitoring plexiform neurofibromas should be determined according to the risk and on an individual basis, after consultation with a multidisciplinary team (Table 1, Table 7). Symptomatic plexiform neurofibromas should be monitored at shorter intervals with regional MRI. When malignancy is suspected based on clinical or imaging (MRI) signs, 18F-fluorodeoxyglucose positron emission tomography magnetic resonance imaging (^18^FDG PET MRI) (preferred) or 18F-fluorodeoxyglucose positron emission tomography computed tomography (^18^FDG PET CT) (if ^18^FDG PET MRI is not available) combined with regional MRI is advised. In case of a suspected ANNUBP or MPNST, primary resection is recommended when safe and feasible. Otherwise, radiologically (preferably ^18^FDG PET MRI) guided diagnostic biopsy should be performed. Additionally, screening for breast cancer in patients with NF1, preferably with annual breast MRI, should begin at 30 years (Table 1, Table 11). When MRI is not available mammography can be used.

**Table 8:** Guideline Recommendations for orbital and periorbital plexiform neurofibroma.

Orbital and periorbital plexiform neurofibroma		
No	Recommendations	Strength
1	The clinical assessment of patients with NF1 suspected of having an orbital and periorbital plexiform neurofibroma, should be physical examination looking for blepharoptosis, proptosis, eyelid oedema, orbital dysplasia and/or dystopia, distortion of the (peri)orbital skeleton, pulsation of the eye, and strabismus. Clinical testing of vision and refractive error, visual field, ocular motility and alignment, and evaluation of the optic disc to exclude glaucoma or optic neuropathy should be basic steps in the examination of patients with NF1 who are suspected of having an orbital and periorbital plexiform neurofibroma.	strong
2	MRI of the brain and orbits should be performed in all children with a suspected orbital and periorbital plexiform neurofibroma. High-resolution MRI sequences with and without contrast should be acquired through the orbit, face, and cavernous sinus. Whenever possible the radiation exposure from CT scans should be avoided in all children with NF1.	strong
3	Symptomatic clinical progression, of known orbital and periorbital plexiform neurofibromas, and new findings should be the primary indication for imaging assessment and follow-up, and this should be by MRI.	strong
4	Given the burden of visible manifestation in patients with NF1 with orbital and periorbital plexiform neurofibroma, people with orbital and periorbital plexiform neurofibroma should be offered psychological support in decisions of management (please see recommendations in the psychosocial needs, Table 15).	weak

Note. NF1 = Neurofibromatosis type 1; MRI = magnetic resonance imaging; CT = computed tomography.

**Table 9:** Guideline Recommendations for malignant peripheral nerve sheath tumour and atypical neurofibromatous neoplasm of uncertain biologic potential.

Malignant peripheral nerve sheath tumour and atypical neurofibromatous neoplasm of uncertain biologic potential		
No	Recommendations	Strength
1	The following groups of people with NF1 should be considered at high risk of MPNST: • *NF1* microdeletion affecting *SUZ12* • missense variants affecting codons 844-848 • previous atypical neurofibromatous neoplasm with uncertain biologic potential (ANNUBP) • high internal tumour load on WB-MRI or large or multiple plexiform neurofibroma in absence of WB-MRI • neurofibromatous neuropathy • previous radiotherapy • a relative with NF1 and MPNST	strong
2	Clinical assessment for MPNST should consist of assessing the following: • Tumour growth: a rapid increase in the size or a change in growth rate or of an existing plexiform neurofibroma. • Pain: new and persistent, nocturnal, substantial pain/pain that is difficult to control. • New motor deficit, sensory deficit associated with any neurofibroma or peripheral nerve. This includes bladder function, bowel disturbance, swallowing problems and breathing difficulty. • Tumour consistency: development of hard nodule in a previously soft plexiform neurofibroma. People with NF1 and any of the above should be investigated for MPNST.	strong
3	When clinical signs and symptoms point towards malignancy (suspicious tumours), investigation should begin with regional MRI. Prior to surgery, MRI should be carried out and ^18^FDG PET MRI (preferred) or ^18^FDG PET CT (if ^18^FDG PET MRI is not available) undertaken, using visual assessment and semiquantitative assessments with a cut-off standardized uptake value.	moderate
4	In case of a suspected ANNUBP or MPNST, primary resection is recommended if it is safe and feasible. Otherwise, radiologically (preferably^18^FDG PET MRI) guided diagnostic biopsy should be performed. This biopsy should be taken at the discretion of a (sarcoma) multidisciplinary team, as tumours can be heterogeneous, with the potential for a false negative result by missing malignant parts of the tumour.	strong
5	There is no place for watchful waiting in MPNST and urgent surgical resection should be the mainstay for treatment (if possible), with post-operative assessment for recurrence.	strong
6	Treatment decisions, on initial surgery and/or (neo)adjuvant chemo- or radiotherapy should be guided by an experienced multidisciplinary team.	moderate
7	If a diagnosis of ANNUBP is proven by biopsy then surgery should be the primary treatment option, if this is possible with acceptable morbidity.	strong
8	If an ANNUBP cannot be resected with acceptable morbidity, initial screening with MRI should be conducted at least every 6 months. In case of tumour growth or increase in symptoms, screening should include ^18^FDG PET MRI (preferred) or ^18^FDG PET CT (if ^18^FDG PET MRI is not available). After an initial clinical assessment, the follow-up interval should be determined by the characteristics of the tumour.	moderate

Note. NF1 = Neurofibromatosis type 1; MPNST = malignant peripheral nerve sheath tumour; ANNUBP = Atypical neurofibromateous neoplasm of uncertain biologic potential; WB-MRI = whole-body magnetic resonance imaging; ^18^FDG PET MRI = 18F-fluorodeoxyglucose positron emission tomography magnetic resonance imaging; ^18^FDG PET CT = 18F-fluorodeoxyglucose positron emission tomography computed tomography; MRI = magnetic resonance imaging.

**Table 10:** Guideline Recommendations for juvenile myelomonocytic leukaemia.

Juvenile myelomonocytic leukaemia		
No	Recommendations	Strength
1	At this time the increased risk for JMML in NF1 is not clear, and is almost certainly <1%. As such specific clinical assessment probably should not be conducted.	moderate
2	Observing juvenile xanthogranulomas in children with NF1 may raise awareness to actively search for other alarming signs of JMML (amongst others hepatosplenomegaly, paleness, abnormal lymph nodes), but should not be considered reason enough for extensive investigations for JMML.	weak

Note. JMML = juvenile myelomonocytic leukaemia; NF1 = Neurofibromatosis type 1.

**Table 11:** Guideline Recommendations for breast cancer.

Breast cancer		
No	Recommendations	Strength
1	Despite there being no evidence of outcome benefits from clinical assessment, education about breast self-examination probably should be conducted as it raises awareness and engagement with clinical centres.	weak
2	Screening with annual breast MRI should be the primary approach, mammography being second best alternative when MRI is not available. Age at commencement of screening in NF1 should begin as soon after the age of 30 years as feasible in the local health system context.	moderate
3	Screening should continue until 50 years after which time, screening should be according to national guidelines for the general population.	moderate
4	Risk-reducing bilateral mastectomy for woman without breast cancer should not be performed in patients with NF1 unless there are substantial additional risk factors such as a family history of breast cancer that would elevate risk into a high-risk category.	moderate

Note. MRI = magnetic resonance imaging; NF1 = Neurofibromatosis type 1.

### MRI as preferred imaging screening

Throughout the guideline the use of MRI is recommended and is the preferred method for imaging screening. Imaging for OPGs with MRI should be performed in people where ophthalmological examination is suggestive for an OPG, and in children older than two years with repeated inconclusive or unreliable ophthalmological exam, e.g. due to age or attention deficit (Table 3).^24^^,^^25^ Currently there are no MRI specific technical recommendations in regard to the imaging sequences to employ in NF1 brain imaging. However, guidance can be sought by combining recommended European Society for Paediatric Oncology (SIOPE) brain imaging guidelines^26^ and Response Assessment in Paediatric Neuro-Oncology (RAPNO) guidelines for imaging of low-grade tumours,^27^ including imaging of the optic tract. Using WB-MRI the number, location and overall tumour burden can be assessed, without exposing the patient to any x-ray (gamma) radiation.^28^^,^^29^ Since the population with NF1 already has an increased risk of developing cancer, limiting diagnostic radiation exposure in this population is recommended.^30^ Therefore, the use of CT should be avoided in children with NF1 when possible and when used should be carefully considered. It is probably also safer not to use mammography for breast cancer screening, although the radiation exposure is low.^31^

### Optic pathway glioma and low-grade glioma

Low-grade gliomas (LGGs) are the most common central nervous system tumours in individuals with NF1 and have been reported in approximately 20% of all patients with NF1.^32^ The vast majority of these gliomas are diagnosed in children before the age of eight years (mean age at diagnosis is 4.5 years) and originates within the optic nerves, optic tract or optic chiasm (optic pathway gliomas: OPGs (Table 3)).^33^ The incidence of OPGs is as high as 15% in children with NF1 and 66–75% of all central nervous system tumours found in this population are OPGs.^24^^,^^33^^,^^34^ About 40–50% of children develop symptoms due to their OPG, but only 15–20% of all patients with OPG are treated due to progressive symptoms.^35^, ^36^, ^37^, ^38^, ^39^ Symptoms may include visual signs such as squint or strabismus, visual loss, proptosis, papilledema and nystagmus as well as precocious puberty or raised intracranial pressure.^40^ Any patient with NF1 diagnosed with an asymptomatic or symptomatic OPG should receive a referral to an expert unit for the management of this NF1 associated OPG (e.g. paediatric, ophthalmology, and/or neuro-oncology) (Table 3). According to the Response Evaluation in Neurofibromatosis and Schwannomatosis (REiNS) criteria, visual acuity (VA) is the best objective, reliable and functional outcome to assess the visual impact of an OPG and the response to treatment.^41^ Brain imaging (including orbits) is clearly indicated in children with visual symptoms and when ophthalmologic assessment is not reliably feasible, e.g. due to age or attention deficit.^24^^,^^25^ Regular ophthalmological examination of children with NF1 is recommended (Table 3).^42^ OCT is increasingly available in routine diagnostics and can be an objective measure of retinal nerve fibre layer (RNFL) and retinal ganglion cell layer (RGCL) thickness. Changes in RNFL and RGCL thickness over time have been shown to correlate with visual function in children with NF1 associated OPGs.^25^ Thus, OCT has emerged as potential biomarker for visual (dys-) function. However, OCT is not readily available yet and requires optimal cooperation (for fixed devices) or even sometimes sedation (for handheld devices) in young children.

Patients with NF1 have a high risk of developing LGGs (mainly pilocytic astrocytomas) in locations other than the optic pathway.^43^ Approximately 4–5% of all individuals with NF1 presents with non-OPG brain gliomas.^32^^,^^44^ The vast majority of the non-OPG tumours in NF1 are LGGs developing in childhood; however, they tend to arise somewhat later than OPGs (mean age seven years). The childhood non-OPGs (Table 4) are primarily located in the basal ganglia, thalamus, cerebellum and brainstem but can also be seen in the cerebral hemispheres and spinal cord.^45^, ^46^, ^47^ LGGs can be seen concurrently with OPGs in children (50%–60%).^46^^,^^48^ As most LGGs develop in childhood,^46^^,^^49^ adults with NF1 are more likely to develop high-grade gliomas (HGGs), and even rarely glioblastomas (GBMs) (Table 5).^48^ Predictors for treatment include: symptomatic tumours; thalamic, cerebellar and frontal location; multiple and diffuse lesions.^45^ When feasible, complete resection of the lesion is the best therapeutic option for a symptomatic or progressive LGG (Table 4, Table 5).^50^ Asymptomatic LGGs and those with mild symptoms can be followed over time with imaging, a watch-and-wait approach is recommended.^45^^,^^46^ For inoperable symptomatic gliomas needing treatment, chemotherapy is recommended, and radiotherapy should be avoided.^50^

### Neurofibromas: cutaneous neurofibroma, plexiform neurofibroma, orbital and periorbital plexiform neurofibroma and atypical neurofibromatous neoplasm of uncertain biologic potential

Neurofibromas, benign peripheral nerve sheath tumours, are the hallmark manifestation of NF1. Cutaneous neurofibromas are tumours that originate from within the skin. More than 95% of the NF1 population will develop cutaneous neurofibromas during their lifetime.^51^ Cutaneous neurofibromas typically start developing in puberty and increase in number throughout life.^52^ Although they are not life threatening, they do have a negative impact on the quality of life and can cause significant discomfort and or disfigurement.^53^^,^^54^ Due to this discomfort or aesthetic burden, cutaneous neurofibromas may be treated/removed by laser, surgery, electrodessication or radiofrequency ablation (Table 6).^55^, ^56^, ^57^, ^58^, ^59^, ^60^, ^61^

Plexiform neurofibromas are benign, diffuse or nodular growing tumours of the nerve sheath arising in approximately 40–60% of patients with NF1, depending on the use of WB-MRI to identify clinically undetectable plexiform neurofibromas (Table 7)^29^^,^^62^^,^^63^ Plexiform neurofibromas involving the eyelid, orbit, and periorbital structures have been labelled orbital and periorbital plexiform neurofibromas and have an incidence of less than 10% in children with NF1.^64^ Orbital and periorbital plexiform neurofibromas frequently cause vision loss secondary to deprivational or anisometropic amblyopia as well as glaucoma (Table 8).^64^ Orbital and periorbital plexiform neurofibromas usually cause aesthetic disfigurement and functional impairment.^65^

At transition from childhood to adulthood, brain and WB-MRI is recommended to screen for possible brain tumours and to evaluate the internal plexiform neurofibroma tumour burden respectively (Table 1, Table 5, Table 7).^29^^,^^66^ Surgery should be considered for symptomatic plexiform neurofibromas, and increased monitoring is recommended.^67^^,^^68^ For symptomatic, inoperable symptomatic plexiform neurofibromas in patients with NF1 MEK inhibitors may be considered.^69^ Plexiform neurofibromas are associated with an increased risk for malignant transformation to MPNSTs, and an intermediate (premalignant) stage between plexiform neurofibromas and MPNSTs exists, called ANNUBP.^70^^,^^71^ Almost half of the ANNUBPs are palpable lesions and about 80% cause clinical symptoms (Table 9).^72^ In case of a suspected ANNUBP or MPNST, primary resection is recommended if safe and feasible. Otherwise, radiologically (preferably ^18^FDG PET MRI) guided diagnostic biopsy should be performed. This biopsy should be taken at the discretion of a (sarcoma) multidisciplinary team, as tumours can be heterogeneous, with the potential for a false negative result by missing malignant parts of the tumour (Table 9).^71^^,^^72^ Early detection and resection of these premalignant lesions seems to prevent further malignant transformation as subtotal resection of ANNUBPs did not result in recurrence (Table 9).^73^^,^^74^

### NF1 associated malignancies: malignant peripheral nerve sheath tumour, high-grade glioma, juvenile myelomonocytic leukaemia and breast cancer

The typical malignant manifestations associated with NF1 include MPNSTs, HGGs, juvenile myelomonocytic leukaemia (JMML) and breast cancer. Patients with NF1 have an 8–16% lifetime risk of developing MPNSTs at any age; however, it is usually diagnosed between the age of 20–40 years.^16^^,^^47^^,^^65^^,^^72^^,^^75^, ^76^, ^77^ Some individuals with NF1 have an elevated risk for MPNST development (these individuals are specified in Table 9). Clinical assessment should include screening for the following clinical features suggestive for a MPNST: rapid tumour growth, new and persistent, substantial pain, new motor deficit/weakness, sensory deficit, sphincter disturbance, swallowing or breathing difficulty, or any changes in tumour consistency (Table 9). MPNSTs can metastasize widely and are associated with a lower overall survival in NF1 compared to MPNSTs in the general population.^77^^,^^78^ Most MPNSTs develop from pre-existing plexiform neurofibromas and are difficult to differentiate from ANNUBPs.^66^^,^^72^^,^^79^ The combination of regional MRI and ^18^FDG PET helps identifying lesions with a high suspicion for malignancy (Table 1, Table 9).^80^ In some cases, guided biopsy will be needed prior to excision, for a diagnosis (Table 9). The mainstay of treatment for MPNSTs is complete excision with wide margins; however, chemotherapy and radiotherapy might be considered in specific situations (Table 9).^73^^,^^74^

HGGs are mainly observed in adults and arise most frequently in the cerebral hemispheres. The course of HGGs in adults with NF1 is more aggressive than their LGG counterparts. Compared to the general population the risk of developing a HGG is 50-fold in the NF1 population; however, adults with NF1 associated HGGs have a better prognosis than individuals with sporadic HGGs.^81^^,^^82^ HGGs contribute to the increased mortality rate in patients with NF1.^18^^,^^83^ Imaging screening for gliomas should be considered at the age of transition from childhood to adulthood for all patients with NF1, and when new symptoms arise suggestive of a brain tumour (Table 1, Table 5). Imaging should take the form of a tumour protocol brain MRI including contrast enhancement if clinically justifiable (Table 5).^44^^,^^45^ Due to the major differences between the NF1 and non-NF1 brain tumours regarding natural history, prognosis, underlying molecular mechanisms and disease manifestation there are currently no NF1-specific treatments for HGGs.^84^ The poor prognosis and increased mortality rate for patients with NF1 associated HGGs highlight the need for better treatment options.

JMML is a rare type of leukaemia with a higher incidence in children with NF1. Although JMML is not a frequent complication of NF1, patients with NF1 are largely overrepresented in the group of children with this leukaemia type.^20^^,^^83^^,^^85^^,^^86^ It has been suggested that children with NF1 with juvenile xanthogranulomas have an increased risk for JMML. However, a retrospective comparative register study did not find an increased risk for malignancy associated with juvenile xanthogranulomas.^87^ Since JMML is rare in individuals with NF1, specific clinical assessment for JMML is not advised in children with NF1 and juvenile xanthogranulomas (Table 1, Table 10).

An increased incidence of breast cancer has been reported in females with NF1.^88^, ^89^, ^90^, ^91^, ^92^, ^93^, ^94^ Interestingly the increased incidence of breast cancer was limited to women between the age of 30 and 50 years, while matching the overall increased breast cancer risk for women above the age of 50. NF1 associated breast cancer was more often oestrogen receptor negative, progesterone receptor negative and human epidermal growth factor receptor 2 (HER2) positive, which are all factors correlated with a poor prognosis.^92^ Annual screening for breast cancer in females with NF1 is advised to start at the age of 30 years preferably by breast MRI (Table 1, Table 11). From the age of 50 years females with NF1 should be screened for breast cancer according to the national guidelines for population screening in their country (Table 1, Table 11).

### Other NF1 associated tumours: phaeochromocytoma and paraganglioma, *glomus tumour* of the digits and gastrointestinal stromal tumour

Other oncological manifestations associated with NF1 include phaeochromocytoma, paraganglioma, glomus tumour of the digits and GIST. In cross sectional studies of adults with NF1, phaeochromocytoma and paraganglioma have been reported to be present in 1–5% of patients with NF1 at a median age of 40–50 years at diagnosis.^16^^,^^95^, ^96^, ^97^ Many phaeochromocytomas and paragangliomas are found incidentally.^96^^,^^98^ It has been reported that patients with NF1 have an increased risk to develop malignant phaeochromocytoma and paraganglioma compared to sporadic cases (12% vs 4%).^96^ About 50% of phaeochromocytomas and paragangliomas become symptomatic, typical symptoms being headache, palpitations, sweating or high blood pressure.^97^ Biochemical testing for secreting phaeochromocytoma and paraganglioma is recommended when suggestive symptoms are present or during pregnancy and before elective surgery requiring general anaesthesia. Biochemical testing includes the measurement of urinary or plasma catecholamines and metanephrines.^97^^,^^99^ However, routine biochemical screening for phaeochromocytoma and paraganglioma in adults with NF1 is not recommended at this moment (Table 1, Table 12). For biochemically active lesions laparoscopic cortical sparing adrenalectomy should be considered (Table 12).^99^

**Table 12:** Guideline Recommendations for phaeochromocytoma and paraganglioma.

Phaeochromocytoma and paraganglioma		
No	Recommendations	Strength
1	Routine biochemical screening for phaeochromocytoma and paraganglioma is not recommended in people with NF1 except for all women with NF1 who are contemplating pregnancy or are already pregnant.	moderate
2	Biochemical testing for phaeochromocytoma and paraganglioma should be conducted in any person with NF1 who has raised blood pressure unexplained by other medical reason.	moderate
3	Biochemical testing for phaeochromocytoma and paraganglioma might be considered prior to any elective surgical procedures requiring general anaesthesia in adult patients with NF1.	weak
4	As in any phaeochromocytoma and paraganglioma predisposition syndrome surgery should be considered for symptomatic or biochemically active lesions.	strong
5	A cortical-sparing adrenalectomy should be the preferred approach due to the risk of metachronous contralateral adrenal tumour.	moderate

Note. NF1 = Neurofibromatosis type 1.

Glomus tumours of the digits are small benign tumours of the glomus body located in the thermoregulatory apparatus of the fingers and toes.^3^^,^^100^ The typical symptoms include localised tenderness, severe paroxysmal pain and sensitivity to cold (Table 1, Table 13).^100^ Treatment consists of excision under local anaesthesia (Table 13). Patients with NF1 are more likely to present with multifocal tumours, and recurrence after surgical resection was also reported in NF1 GTs.^100^, ^101^, ^102^, ^103^ Glomus tumours of the digits are often under-recognized, thereby delaying the diagnosis and leading to chronic pain in patients.

**Table 13:** Guideline Recommendations for glomus tumours of the digits.

Glomus tumours of the digits		
No	Recommendations	Strength
1	Glomus tumours of the digits are easily missed and therefore clinical suspicion is essential to make a diagnosis of glomus tumours of the digits. Clinical diagnosis should be based on patient reported typical symptoms (see recommendation 2) and on visual examination of the nail beds and palpation.	moderate
2	The majority of people will have at least two of the following symptoms: localised tenderness, severe paroxysmal (lancinating, similar to being hit on the nailbed) pain and sensitivity to cold. Visual inspection may show purplish discolouring of the nailbed.	moderate
3	Glomus tumours of the digits occur mostly in adulthood, but should also be considered in children/adolescents with typical symptoms.	weak
4	Surgical excision should be considered for painful glomus tumours of the digits.	moderate

It has been estimated that patients with NF1 have a 200-fold increased risk of developing GISTs compared to the general population.^104^ Patients with NF1 typically manifest GISTs at middle age (mean age 52.8 years) and tend to develop multiple GISTs, frequently located in the small intestine.^104^, ^105^, ^106^, ^107^ GISTs can cause abdominal pain, severe intestinal bleeding and intestinal (sub)obstruction. Screening for GISTs is recommended if suggestive symptoms are present (Table 1, Table 14). Standard practice for symptomatic GISTs is surgical resection with wide margins and follow-up with MRI (or CT abdomen if MRI is not possible) (Table 14).^108^^,^^109^ If incidentally detected by MRI, resection of asymptomatic GISTs with a diameter of 2 cm or more is recommended. Smaller asymptomatic lesions may be monitored by abdominal MRI (Table 14). NF1-related GISTs typically lack alterations in *KIT* or *PDGFRA,* seen in sporadic GISTs, resulting in unresponsiveness to therapy with tyrosine kinase inhibitors.^104^^,^^105^^,^^107^^,^^108^^,^^110^

**Table 14:** Guideline Recommendations for gastrointestinal stromal tumours.

Gastrointestinal stromal tumours		
No	Recommendations	Strength
1	Investigation for GIST should only be conducted if there is clinical suspicion.	moderate
2	Clinical suspicion should be raised in the presence of gastrointestinal discomfort, weight loss, anaemia, gastrointestinal bleeding, abdominal pain, palpable abdominal mass, or intestinal obstruction.	moderate
3	Resection should be considered for at least large (>2 cm) or symptomatic tumours as there is a risk for bleeding and rupture and risk for malignancy with metastasis.	strong
4	People with an incidentally detected GIST that is asymptomatic AND <2 cm diameter should be monitored at least once a year with abdominal MRI (or CT abdomen if an MRI not possible), for at least 5 years, and thereafter to be performed every 2 years.	moderate

Note. GIST = gastrointestinal stromal tumour; MRI = magnetic resonance imaging; CT = computed tomography.

### Psychosocial needs

NF1 is a lifelong condition with a major impact on the quality of life and mental health of the patient and their family.^4^^,^^111^, ^112^, ^113^, ^114^, ^115^ Individuals with NF1 may suffer from chronic pain (often associated with tumours), fatigue (unknown cause) and low self-esteem due to visible disfigurement (from cutaneous neurofibromas and plexiform neurofibromas). The constant fear of developing malignancy, treatment interventions and increasing tumour burden has severe, negative effects on their functional and mental wellbeing.^116^, ^117^, ^118^, ^119^ In addition, other common difficulties associated with NF1 such as learning disabilities and behaviour problems impair their emotional, social and cognitive skills. Hence, these patients need an even wider support network during and after their tumour management. The stress caused by this condition not only affects the patients but also their family, friends and caregivers. The impact of NF1 as a cognitive and behavioural disorder, suggests guidance in the following areas: the importance and timescales of psychosocial and neuropsychological assessment in NF1; psychoeducation; and more tailored psycho-social interventions. Additional to NF1 related tumour treatment and management, regular screening for psychosocial wellbeing and neuropsychological functioning is strongly advised (Table 1, Table 15). To ensure this, it is strongly advised to have a psychologist as a member of the multidisciplinary team, to support patients and families when making decisions about diagnosis, management and treatment (Table 15).

**Table 15:** Guideline Recommendations for psychosocial needs.

Psychosocial needs		
No	Recommendations	Strength
1	NF1 has a significant effect on psychosocial and neuropsychological functioning and impacts on quality of life. It is strongly advised to have a psychologist as a member of the multidisciplinary team, to support patients and families when making decisions about diagnosis, management and treatment.	weak
2	Psychosocial wellbeing and neuropsychological functioning should be addressed at each clinic visit. These may include assessing e.g. anxiety and depression, coping mechanisms and patient reported outcomes.	weak
3	The information and guidance for patients with NF1 and family members should be age-appropriate and tailored to the needs of the individual, potential interventions to reduce the impact of NF1 on psychosocial functioning and quality of life should be included.	weak

Note. NF1 = Neurofibromatosis type 1.

## Discussion

The goal of tumour surveillance is to detect neoplasms before they become symptomatic, and for interventions to have a better chance of being curative or preventative of functional impairment. The proposed recommendations for tumour surveillance for NF1 require a coordinated multidisciplinary approach and significant patient commitment. The guidelines apply to all individuals with NF1. Where appropriate, we adapted recommendations to different age groups, individuals with different types of pathogenic variants in *NF1* or to pre-existing medical history that might influence risks for a specific tumour type in NF1. Given age dependent risks, specific recommendations for children versus adults are provided in the guidelines. Furthermore, evidence for age specific recommendations is based on the underlying patient populations in existing studies, rather than on a clearly defined threshold. We used the term ‘children’ or ‘childhood’ for ages 0–16 years and ‘adults’ or ‘adulthood’ for ages of 18 and older, with a variable transition from childhood to adulthood between 16 and 18 years as applicable to local health care settings. Finally, we discuss the psychosocial support for people with NF1 during tumour surveillance and management. We tackled the issues of living with uncertainty of developing a tumour, during monitoring of a tumour or during and after treatment of a tumour.

While developing the guideline, gaps requiring further research have been identified. In general, the highly variable course of NF1 is well recognised, but detailed knowledge on the course of some NF1 manifestations is lacking. Well documented data from (prospective) multicentre longitudinal cohorts including biomarkers, genotype–phenotype associations and advanced imaging studies will therefore be necessary to improve future guidelines. There is a need for new paradigms for modelling the course of disease by integrating data from imaging, clinical examination, mutational analysis and (non-invasive) biomarker data. Such modelling will allow us to define which patients are at high risk for malignant tumours and need specific screening; and may also help identify responses to new targeted therapies to improve survival in specific subgroups of individuals with NF1. An international multicentre NF1 registry including longitudinal natural history data regarding NF1-associated tumours is essential, but challenging and a task for institutions dedicated to rare diseases. Sufficient evidence is not always available concerning the timing and intervals of routine surveillance for specific tumours in NF1. The guideline group underlined the need for increased monitoring of NF1 during transition age to screen for plexiform neurofibromas, ANNUBPs and its’ potential transition to MPNSTs in adulthood. This is also supported by a study among parents and adolescents with NF1.^118^ However, future studies exploring the need and expected effects of increased screening on outcome are highly needed.

In the current guideline WB-MRI and cerebral MRI are introduced as a screening modality. Although there is evidence that this approach will detect peripheral nerve sheath tumours requiring further monitoring in adulthood, the question remains whether standard and interval scanning will prevent neurological deficit, prolong life and/or will give a better patient outcome. There is even less evidence to recommend brain MRI during transition to adulthood. Unnecessary (harmful) interventions and uncertainty/stress in patients with NF1 may even result in a perceived worsened patient outcome and quality of life. Conversely, fixed and comprehensive examination intervals (e.g. WB-MRI, which is not routinely available in many health care systems in the EU) can provide guidance and safety for both healthcare professionals and patients. There is a need to study the effects of the implementation of this guideline on patient reported outcome measures (PROMs). Specifically for cutaneous neurofibromas, we encourage further research in (patient reported) outcome measures. Currently we focus on single symptoms in small sub-cohorts (e.g. symptomatic or non-operable plexiform neurofibromas), but including more parameters (e.g. cognition) will inform on effects or side effects of treatment on other manifestations in NF1. As more treatment options become available in the future, it is necessary to define indications for specific therapies. This applies also to current available therapies, and this position may change with upcoming innovative therapeutic options. This in particular requires the expertise of a multidisciplinary team in NF1. In addition, the upcoming therapeutic options for manifestations in NF1 may favour screening to identify patients in early stages for such treatment approaches. In the current guideline, recommendations for psychological support are mainly based on general studies, only few exist for NF1 specific populations. Future studies may define the content and type of psychological support and evaluate its impact on patients. It has been demonstrated that body image is an important link between disease visibility and psychological well-being in patients with NF1. Development of adequate PROMs will help to evaluate psychotherapeutic interventions to improve body image in NF1.

Patient education is an important aspect of tumour surveillance. Specifically, there is a need to improve our knowledge on how to educate and guide people with cognitive deficits or problems in social skills, symptoms of attention-deficit hyperactivity disorder and autism spectrum disorder about the risks of tumours and their potential management.

This guideline has been developed by a convenience sample of NF1 experts and by a Delphi approach. In the selection of experts we tried full coverage of European countries and medical specialty. Two advisors from the USA have given feedback on the draft recommendations and evidence summaries, but were excluded to participate in the Delphi survey. As the selection process of experts is initiated by members of ERN GENTURIS, this might have caused little bias. The Delphi approach provided us two key benefits, i.e. the involvement of large numbers of participants from all over Europe without face to face contact,^120^ and it avoids the possible dominance of particular individuals by reaching consensus through anonymity and the use of all answers when evaluating the results.^121^ A limitation is that the Delphi method has no standard method for defining consensus; we used a threshold of >60% to define agreement, although for most recommendations we achieved a consensus of >85% agreement.

In this guideline, we defined recommendations for tumour management in NF1, balancing appropriate care for those in need versus unnecessary treatments for those without complications. We also incorporated tumour related psychosocial and quality of life aspects. The guidelines are not meant to be prescriptive and may be adjusted according to the local health care system. Given the low prevalence of the condition, its many potential manifestations and rare complications, decisions about management should always include discussion with the local multidisciplinary teams including an NF1 experts.

## Contributors

The ERN GENTURIS NF1 Tumour Management Guideline Group consisted of Joan Brunet, Frank Van Calenbergh, Catherine Cassiman, Prof. Dr Thomas Czech, María José Gavarrete de León, Henk Giele, Susie Henley, Conxi Lazaro, Vera Lipkovskaya, Eamonn Maher, Vanessa Martin, Irene Mathijssen, Enrico Opocher, Ana Elisabete Pires, Thomas Pletschko, Eirene Poupaki, Vita Ridola, Andre Rietman, Thorsten Rosenbaum, Alastair Santhouse, Astrid Sehested, Ian Simmons, Walter Taal, and Anja Wagner, including the Core Working Group consisting of Charlotte Carton, D. Gareth Evans, Ignacio Blanco, Reinhard E. Friedrich, Rosalie E. Ferner, Said Farschtschi, Hector Salvador, Amedeo A. Azizi, Victor Mautner, Eric Legius, Claas Röhl, Sirkku Peltonen, Stavros Stivaros, and Rianne Oostenbrink. The Core Working Group was primarily responsible for drafting subsections of the guideline full text underlying the manuscript, and for developing and editing the recommendations. They agreed upon the final recommendations after the modified Delphi approach. Members of the Tumour Management Guideline Group reviewed text and recommendations of the guideline underlying the manuscript. Charlotte Carton and Rianne Oostenbrink were primarily responsible for the writing of the manuscript; all members of the Core Working Group were involved in outlining the structure of the manuscript and reviewed and commented previous versions of the manuscript. The ERN GENTURIS NF1 Tumour Management Guideline Group, including the Core Working Group as well as the other Delphi participants listed in the acknowledgements participated in the modified Delphi. All listed authors commented on drafts and agreed upon the final manuscript.

## Data sharing statement

Not applicable to the approach underlying the manuscript.

## Declaration of interests

All members of the ERN GENTURIS NF1 Tumour Management Guideline Group, including the Core Working Group, have provided disclosure statements on all relationships that they have that might be perceived to be a potential source of a conflict of interest. Dr. Azizi reports personal fees from 10.13039/100004325AstraZeneca, personal fees from Hofmann, LaRoche, outside the submitted work. Dr. Blanco reports personal fees from AstraZeneca, outside the submitted work. C. Carton reports grants from EU-PEARL, the innovative Medicines Inititaive 2 Joint Undertaking under grant agreement no 853966, outside the submitted work. Dr. Evans reports personal fees from AstraZeneca, personal fees from Springworks Therapeutics, outside the submitted work. Dr. Farschtschi has nothing to disclose. Dr. Ferner reports grants and personal fees from AstraZeneca, outside the submitted work. Dr. Friedrich has nothing to disclose. Dr. Legius reports personal fees from AstraZeneca, personal fees from Springworks Therapeutics, grants from EU-PEARL, the innovative Medicines Inititaive 2 Joint Undertaking under grant agreement no 853966, outside the submitted work. Dr. Mautner has nothing to disclose. Dr. Oostenbrink reports personal fees from 10.13039/100004325AstraZeneca, personal fees from 10.13039/100004325AstraZeneca, grants from EU-PEARL, the innovative Medicines Inititaive 2 Joint Undertaking under grant agreement no 853966, outside the submitted work. Dr. Peltonen has nothing to disclose. C. Röhl reports grants and personal fees from 10.13039/100004336Novartis, grants from 10.13039/100004325AstraZeneca, grants from 10.13039/100004337Roche, grants and personal fees from 10.13039/100004319Pfizer, personal fees from Boehringer, personal fees from ingelheim, outside the submitted work. Dr. Salvador Hernandez reports personal fees from 10.13039/100004325AstraZeneca, outside the submitted work. Dr. Stivaros has nothing to disclose. Dr. Brunet reports personal fees from 10.13039/100004325AstraZeneca, outside the submitted work. Dr. Cassiman has nothing to disclose. Dr. Czech has nothing to disclose. M. J. Gavarrete de León has nothing to disclose. Dr. Giele has nothing to disclose. Dr. Henley has nothing to disclose. Dr. Lazaro reports personal fees from 10.13039/100004325AstraZeneca, outside the submitted work. V. Lipkovskaya has nothing to disclose. Dr. Maher reports other from Illumina and MSD, outside the submitted work. V. Martin has nothing to disclose. Dr. Mathijssen has nothing to disclose. Dr. Opocher reports personal fees from 10.13039/100004325AstraZeneca, outside the submitted work. A. E. Pires has nothing to disclose. Dr. Pletschko has nothing to disclose. Dr. Ridola has nothing to disclose. Dr. Rietman has nothing to disclose. Dr. Rosenbaum reports personal fees from 10.13039/100004325AstraZeneca, other from Lonza AG, outside the submitted work. Dr. Santhouse has nothing to disclose. Dr. Sehested has nothing to disclose. I. Simmons has nothing to disclose. Dr. Taal reports grants from 10.13039/100004336Novartis, outside the submitted work. Dr. Van Calenbergh has nothing to disclose. Dr. Wagner has nothing to disclose.
