# Supplementary material for: ERN GENTURIS tumour surveillance guidelines for individuals with neurofibromatosis type 1
Source: eClinicalMedicine. 2023 Jan 13;56:101818. doi: 10.1016/j.eclinm.2022.101818 (PMC9845795; doi:10.1016/j.eclinm.2022.101818)
Supplement: Supplementary material [file mmc1.docx]

**other members of the neurofibromatosis type 1 tumour management guideline group in alphabetical order:**

| Author | Affiliation |
| --- | --- |
| Assoc. Prof. Dr. Joan Brunet | Catalan Institute of Oncology, Barcelona, Spain; Full Member of the European Reference Network on Genetic Tumour Risk Syndromes (ERN GENTURIS). |
| Prof. Dr. Frank Van Calenbergh | University Hospital Leuven, Campus Gasthuisberg, Leuven, Belgium; Full Member of the European Reference Network on Genetic Tumour Risk Syndromes (ERN GENTURIS). |
| Dr Catherine Cassiman | University Hospital Leuven, Campus Gasthuisberg, Leuven, Belgium; Full Member of the European Reference Network on Genetic Tumour Risk Syndromes (ERN GENTURIS). |
| a.o. Prof. Dr Thomas Czech | Medical University of Vienna, Austria; Affiliated Partner of the European Reference Network on Genetic Tumour Risk Syndromes (ERN GENTURIS). |
| María José Gavarrete de León | ACNefi (Associació Catalana de les Neurofibromatosis), Spain |
| Prof. Henk Giele | Oxford University Hospitals Foundation Trust, UK |
| Dr. Susie Henley | Neurofibromatosis Centre, Department of Neurology, Guy’s and St Thomas’ NHS Foundation Trust, London, UK |
| Dr. Conxi Lazaro | Catalan Institute of Oncology, ICO-IDIBELL-ONCOBELL, Barcelona, Spain; Full Member of the European Reference Network on Genetic Tumour Risk Syndromes (ERN GENTURIS).  Centro de Investigación Biomédica en Red de Cáncer (CIBERONC), Spain |
| Vera Lipkovskaya | NF patient organisation 22/17, Belgium |
| Prof. Eamonn R Maher | University of Cambridge, UK  Supporting partner of the European Reference Network on Genetic Tumour Risk Syndromes (ERN GENTURIS). |
| Vanessa Martin | Childhood Tumour Trust, UK |
| Prof. Irene Mathijssen, MD, PhD | Erasmus Medical Center, Rotterdam, The Netherlands; Full Member of the European Reference Network on Genetic Tumour Risk Syndromes (ERN GENTURIS). |
| Dr. Enrico Opocher | Padua University Hospital, Padua, Italy ; Full Member of the European Reference Network on Genetic Tumour Risk Syndromes (ERN GENTURIS).  Great Ormond Street Hospital, London, UK |
| Ana Elisabete Pires | Associação Portuguesa de Neurofibromatose  BIOPOLIS/CIBIO-InBIO, Universidade do Porto, Portugal |
| Dr. Thomas Pletschko | Medical University of Vienna, Austria; Affiliated Partner of the European Reference Network on Genetic Tumour Risk Syndromes (ERN GENTURIS). |
| Eirene Poupaki | NF PANHELLENIC ASSOCIATION “LIFE WITH NF”, Greece |
| Dr. Vita Ridola | MITERA Children’s Hospital, Athens, Greece |
| Andre Rietman, MD, PhD | ErasmusMC, Rotterdam, The Netherlands; Full Member of the European Reference Network on Genetic Tumour Risk Syndromes (ERN GENTURIS). |
| Prof. Dr. Thorsten Rosenbaum | Sana Kliniken Duisburg, Universitat Duisburg-Essen, Germany |
| Dr. Alastair Santhouse | South London & Maudsley NHS Foundation Trust, London, UK |
| Astrid Sehested, MD | Rigshospitalet, Copenhagen, Denmark; Full Member of the European Reference Network on Genetic Tumour Risk Syndromes (ERN GENTURIS). |
| Ian Simmons | Leeds Teaching Hospitals NHS Trust, UK |
| Walter Taal, MD, PhD | Dep of Neurology / Neuro-Oncology, ErasmusMC Cancer Institute, Rotterdam, The Netherlands; Full Member of the European Reference Network on Genetic Tumour Risk Syndromes (ERN GENTURIS). |
| Anja Wagner, MD, PhD | ErasmusMC Cancer Institute, Rotterdam, The Netherlands; Full Member of the European Reference Network on Genetic Tumour Risk Syndromes (ERN GENTURIS). |
